# Ivermectin and COVID-19

**DOI:** 10.21315/mjms2021.28.2.17

**Published:** 2021-04-21

**Authors:** Viroj Wiwanitkit

**Affiliations:** Department of Community Medicine, DY Patil University, Pune, Maharashtra, India

Dear Editor,

I would like to share ideas on the publication ‘Ivermectin: potential role as repurposed drug for COVID-19’ (1). Dixit et al. (1) discussed ‘the potential for ivermectin to be used as a therapeutic option in COVID-19. In fact, many classic drugs are proposed as potential therapeutic agents against COVID-19. Hydroxychloroquine is a good example (2). Ivermectin, is another potential drug for COVID-19 therapy. In some countries such as India, ivermectin is used for mass chemoprophylaxis of lymphatic filariasis. Nevertheless, there is a high incidence of COVID-19 in the area that ivermectin is routinely used. Therefore, a standard prophylactic dose of ivermectin might not be useful for COVID-19 therapy. Since ivermectin is a classic drug with safety evidence, further trials on ivermectin against COVID-19 are recommended. In some countries such as India, ivermectin is used for mass chemoprophylaxis of lymphatic filariasis (3).
